# Renal Involvement in Linear Nevus Sebaceous Syndrome—An Underrecognized Feature

**DOI:** 10.3390/pediatric13020028

**Published:** 2021-05-01

**Authors:** Chonin Kuok, Kwaiyu Chan

**Affiliations:** Department of Paediatrics, Queen Elizabeth Hospital, Hong Kong, China; chankyw@ha.org.hk

**Keywords:** linear nevus sebaceous syndrome, epidermal nevus syndrome, cystic kidney disease, renal artery stenosis, hypertension

## Abstract

Linear nevus sebaceous syndrome (LNSS) is a rare neurocutaneous disorder. It is characterized by the presence of nevus sebaceous, ocular anomalies, neurological deficits, and convulsion. Renal involvement was not commonly reported. We report a 10-year-old girl with LNSS who had concomitant cystic kidney disease and diffuse aortopathy with bilateral renal artery stenosis, leading to hypertension requiring oral anti-hypertensive medications. The girl presented with chorioretinal coloboma and multiple nevus sebaceous at birth. She had aortic coarctation and received surgical repair at one week of life. She had persistent hypertension during her follow-up. Further investigations were performed to look for causes of hypertension apart from possible re-coarctation. Her magnetic resonance angiogram revealed diffuse aortopathy, which extended from the aortic arch to the abdominal aorta. Branches of the aorta, including the celiac trunk, superior mesenteric arteries, and renal arteries, were also narrowed. Multiple renal cysts were also identified in her right kidney. Interventional angioplasty over the renal arteries was not feasible due to diffuse narrowing of the aorta, especially at the origins of renal arteries. The blood pressure was controlled with oral anti-hypertensive medications. Our case illustrated that pediatricians should be aware of the possible renal involvements in LNSS, which impose a significant impact on the management and long-term prognosis of these patients.

## 1. Introduction

Linear nevus sebaceous syndrome (LNSS) is a rare congenital neurocutaneous disorder, with an estimated incidence of 1 per 10,000 live births [1]. In addition to the classical triad of nevus sebaceous, seizures, and mental retardation, other organ involvements of the ophthalmological, cardiovascular, and skeletal systems have also been reported [2]. However, renal involvement in this syndrome was less prevalent. We report a 10-year-old girl with LNSS with concomitant diffuse aortopathy, bilateral renal artery stenosis, and cystic kidney disease.

## 2. Case Presentation

**Figure 1:**
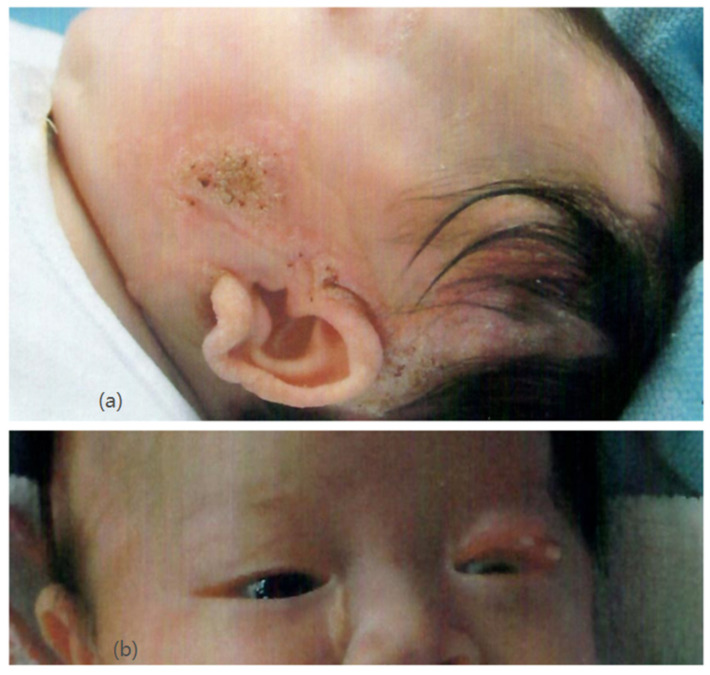
(**a**) (upper) Nevus sebaceous over the left face and scalp. (**b**) (lower) Lipodermoid at the left upper eyelid.

Our patient was an Asian girl who was delivered at 36 weeks of gestation with normal antenatal screening. Her family history was unremarkable. Newborn examination revealed multiple well-defined yellowish plaques over her body, involving her scalp, face, neck, right chest, and upper limb (Figure 1a). Ophthalmological examination showed chorioretinal coloboma in her right eye and a lipodermoid at her left upper eyelid (Figure 1b). She was diagnosed with linear nevus sebaceous syndrome.

An echocardiogram on day four of life showed aortic coarctation at the juxta-ductal region, with the narrowest point of 2 mm, and a 2.5 mm patent ductus arteriosus (PDA) with bidirectional shunt. Surgical repair of the coarctation and division of the PDA were performed on day seven of life. She developed re-coarctation at the repair site and required balloon aortoplasty six weeks after the surgery. At two months old, she also had multiple episodes of focal seizures involving her left limbs. She was put on oral carbamazepine and has remained seizure-free since six years old. The magnetic resonance imaging (MRI) of her brain and spine showed right frontotemporal lobe pachygyria and a lipomatous lesion at the thoracic spinal cord.

She was hypertensive during her follow-up, which was believed to be attributable to her narrowed aortic isthmus demonstrated in her echocardiograms. Her blood pressure was 146/83 mmHg and 137/86 mmHg over her right and left arm, respectively, at seven years old. In view of her persistent hypertension, further investigations were performed to look into other possibilities of hypertension. Her blood tests showed a normal cortisol level = 205 nmol/L (reference: 64–327 nmol/L), free thyroxine T4 level = 11.1 pmol/L (reference: 10.9–19.0 pmol/L), thyroid stimulating hormone = 2.18 mIU/L (reference: 0.35–4.94 mIU/L), and serum creatinine = 46 umol/L (reference: 34–65 umol/L). The plasma renin activity was elevated to 7.11 ng/mL/h (reference: 0.50–5.90 ng/mL/h), and aldosterone level to 1546 pmol/L (reference: < 250 pmol/L). There was no hematuria or proteinuria. The urine catecholamine levels were normal.

**Figure 2:**
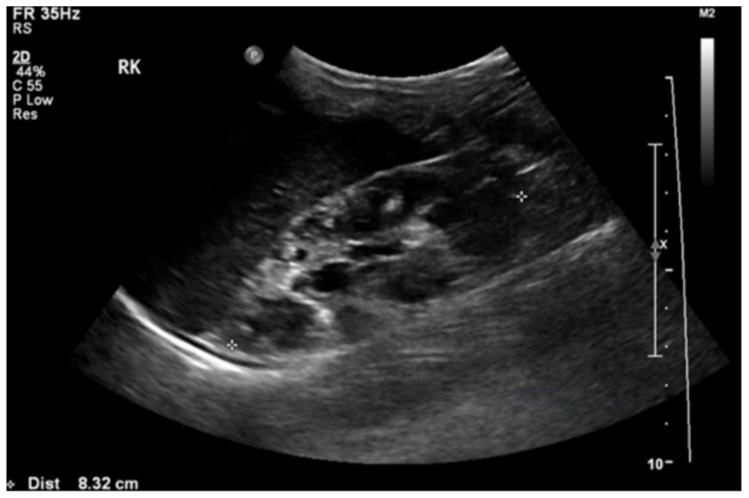
Renal ultrasound showed loss of corticomedullary differentiation of the right kidney with multiple cysts of variable sizes.

A doppler ultrasound revealed focal stenosis at the proximal and mid-portion of the left renal artery, with the raised peak systolic velocity reaching 300 cm/s. There were multiple cysts with no intra-cystic solid component in her right kidney (Figure 2). There were no cysts in the left kidney or liver. Tc99m-MAG3 (99m technetium mercaptoacetyltriglycine) scan showed impaired right renal function with a left-to-right ratio of 68%:32%.

**Figure 3:**
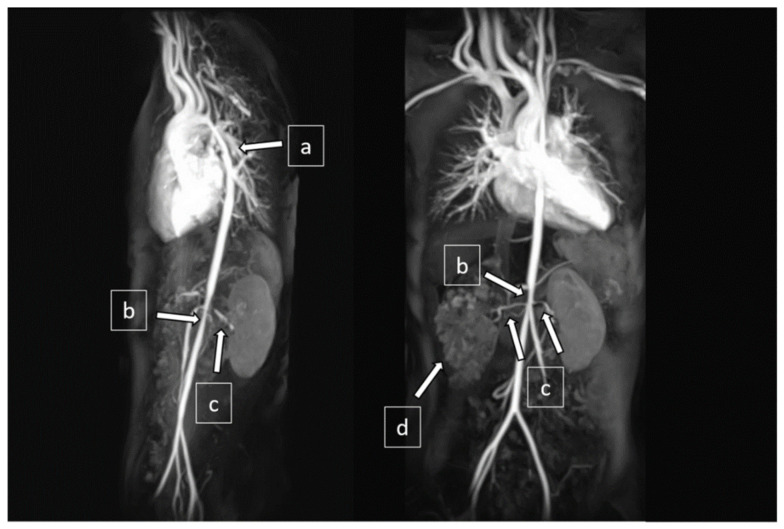
Magnetic resonance angiography (MRA) showed (**a**) narrowing at the distal aortic arch and descending thoracic aorta, (**b**) narrowing at the abdominal aorta, (**c**) bilateral renal artery stenoses, and (**d**) right cystic kidney.

Magnetic resonance angiogram revealed diffuse aortopathy involving the aortic arch (5 mm in diameter at isthmus), thoracic aorta (7 mm), and abdominal aorta (5 mm). The main branches from the aorta were also affected, including bilateral renal artery stenoses (RAS) and narrowed celiac trunk and superior mesenteric artery (2–3 mm in diameter) (Figure 3). The Doppler ultrasound did not show focal stenosis in her common, internal, or external carotid arteries.

Our patient received oral amlodipine and atenolol to control her blood pressure. Interventional angioplasty over the renal arteries was not feasible due to the diffuse narrowing of the aorta, especially near the origins of the renal arteries. She remained asymptomatic, and her blood pressure was controlled at around the 95th percentile for her height. The latest blood tests showed normal serum urea = 4.7 mmol/L (reference: 1.8–6.4 mmol/L) and creatinine level = 47 umol/L (reference: 34–65 umol/L).

## 3. Discussion

Linear nevus sebaceous syndrome (LNSS), also known as Schimmelpenning syndrome, is a sporadic congenital disorder with no gender predilection. It is characterized by craniofacial nevus sebaceous in association with extracutaneous features, including neurological, ocular, and cardiovascular anomalies [2]. The term epidermal nevus syndrome is sometimes used interchangeably to describe the same condition [3]. It was suggested that there are other subtypes of epidermal nevus syndrome besides LNSS, which can be distinguished by different phenotypic and genetic features [2].

Nevus sebaceous is the hallmark of LNSS. It is a type of epidermal nevus that involves hyperplasia of epidermis, hair follicles, and sebaceous and apocrine glands. It typically presents as verrucous, granulated, yellow-orange plaques at birth [4]. The lesions most frequently occur in the scalp in two-thirds of patients, followed by the facial region in one-third [5].

Neurological and ophthalmological manifestations affect 72% and 59% of LNSS patients, respectively [6]. The syndrome is associated with structural brain malformation, including hemimegalencephaly, ventriculomegaly, and pachygyria. Coloboma and choristoma are the common ophthalmological presentations. Other eye involvements include strabismus, retinal anomalies, and vitreous and corneal opacities [7]. Other associations of LNSS, including vitamin D resistant rickets [8], lymphatic malformation [9], and intraspinal lipoma [10], have also been reported.

Our patient presented at birth with distinctive external features of nevus sebaceous over the facial region and body, as well as coloboma and lipodermoid in her eyes. She had neurological abnormalities, including pachygyria, epilepsy, and intraspinal lipoma. In addition to the typical features, our patient also had diffuse narrowing of the aorta with bilateral renal artery stenoses and unilateral cystic kidney disease. The association between nevus sebaceous and renal abnormalities was less described in the literature, possibly due to under-recognition and under-reporting. The previously reported renal associations are summarized in Table 1.

Renal vasculopathy constituted the majority of the abnormalities. Renal artery stenoses were reported in four cases [10,11,12,13]. The stenoses were in conjunction with the narrowing of aorta, which varied in extent and severity. These patients had renovascular hypertension related to the stenoses. Percutaneous renal angioplasty was attempted in one patient, but balloon inflation was unsuccessful [12]. Another patient had a focal renal artery aneurysm with narrowing at the descending thoracic aorta [14]. Oral anti-hypertensive medication was the mainstay of treatment in these patients.

Renal parenchymal involvements were less reported. Nickavar et al. reported a two-year-old boy who had Wilm’s tumor in a non-functioning multi-cystic dysplastic kidney, in addition to skin and neurological manifestations [15]. Bourdeaut et al. reported another patient who had an incidental finding of bilateral polycystic kidneys during a computed tomography (CT) scan [16]. The differential functions of the kidneys and subsequent treatment were not mentioned in this report. In both cases, the renal arteries were not affected. Renal dysplasia and horseshoe kidney were described in other patients [17,18]. The concomitant occurrence of renal vasculopathy and renal cysts in our patient was not previously described.

Most patients with LNSS are recognized at birth due to the distinctive phenotypes. Besides cardiac and neurological complications, physicians should also be aware of possible renal manifestations in these patients, which carry a significant impact on management and long-term prognosis. Blood pressure should be monitored regularly in LNSS patients. Renal doppler ultrasonography should be considered as a non-invasive screening modality for detecting renal structural and vascular abnormalities. Further evaluation by computed tomography angiography or magnetic resonance angiography is warranted in individual cases to delineate the anatomy and extent of the aortopathy for formulating a management plan.

## Figures and Tables

**Table 1:** Summary of the associations between epidermal nevus/nevus sebaceous and renal manifestations.

Authors	Clinical Manifestations			
	Renal	Vascular	Dermatological	Neurological/Ophthalmological
Mall et al. [10]	Left RAS	Hypoplasia of thoracoabdominal aorta	NS extending from mid-face to right parieto-occipital region	Leptomeningeal lipoma Mild mental retardation Exophthalmos, conjunctival pterygium, left corneal opacity
Nagatsuma et al. [11]	Left RAS	Stenoses at abdominal aorta upstream of renal arterial branch, celiac artery, and SMA	NS at the right face and scalp	Right temporal lobe dysplasia, epilepsy Mild intellectual disability Amblyopia, limbal dermoid
Aizawa et al. [12]	Right RAS	Narrowing of abdominal aorta distal to ostium of SMA	EN at the left temporal area, neck, and lower back	Arachnoid cyst Microphthalmos
Alsohim et al. [13]	Bilateral RAS	Stenosis at the transverse arch, left subclavian artery, descending thoracic aorta, celiac trunk, and SMA	NS at the right face and neck	Conjunctival lipodermoid
Juan et al. [14]	Left renal artery focal aneurysm	Stenoses and aneurysms in aortic arch and descending thoracic aorta	NS at the left face and neck	Microphthalmia, coloboma
Nickavar et al. [15]	Wilm’s tumor in unilateral multi-cystic dysplastic kidney	Coarctation of aorta, dilated left iliac and femoral vessels	EN at the chest, trunk, and limbs	Brain atrophic changes, epilepsy
Bourdeaut et al. [16]	Bilateral micropolycystic kidney	Nil	EN along Blaschko line at abdomen	Nil
Almefty et al. [17]	Left renal dysplasia	Coarctation of aorta	NS at the left ear, cheek, and anterior neck	Right hemimegalencephaly, left temporal arachnoid cyst Epibulbar dermoid
Mollica et al. [18]	Horseshoe kidney	Nil	NS at the forehead, right-sided trunk, and limbs	Leptomeningeal hemangioma
Our patient	Bilateral RAS Unilateral cystic kidney disease	Narrowing of aorta involving aortic arch, thoracoabdominal aorta, celiac trunk, and SMA	NS at the scalp, face, neck, right chest, and upper limb	Right frontotemporal pachygyria, epilepsy, intraspinal lipoma Chorioretinal coloboma, lipodermoid

Abbreviations: RAS = renal artery stenosis; NS = nevus sebaceous; EN = epidermal nevus; SMA = superior mesenteric artery.

## Data Availability

All data are reported in the case.
